# Dorsomedial Striatum (DMS) CB1R Signaling Promotes Pavlovian Devaluation Sensitivity in Male Long Evans Rats and Reduces DMS Inhibitory Synaptic Transmission in Both Sexes

**DOI:** 10.1523/ENEURO.0341-24.2024

**Published:** 2025-01-21

**Authors:** Catherine A. Stapf, Sara E. Keefer, Jessica M. McInerney, Joseph F. Cheer, Donna J. Calu

**Affiliations:** ^1^Program in Neuroscience, University of Maryland Baltimore, Baltimore, Maryland 21201; ^2^Department of Neurobiology, University of Maryland School of Medicine, Baltimore, Maryland 21201

**Keywords:** devaluation, dorsal striatum, endocannabinoids, pavlovian conditioning, sex differences

## Abstract

Cannabinoid receptor-1 (CB1R) signaling in the dorsal striatum regulates the shift from flexible to habitual behavior in instrumental outcome devaluation. Based on prior work establishing individual-, sex-, and experience-dependent differences in pavlovian behaviors, we predicted a role for dorsomedial striatum (DMS) CB1R signaling in driving rigid responding in pavlovian autoshaping and outcome devaluation. We trained male and female Long Evans rats in pavlovian lever autoshaping (PLA). We gave intra-DMS infusions of the CB1R inverse agonist, rimonabant, before satiety-induced outcome devaluation test sessions, where we sated rats on training pellets or home cage chow and tested them in brief nonreinforced PLA sessions. Overall, inhibition of DMS CB1R signaling prevented pavlovian outcome devaluation but did not affect behavior in reinforced PLA sessions. Males were sensitive to devaluation while females were not, and DMS CB1R blockade impaired devaluation sensitivity in males. Because these results suggest DMS CB1R signaling supports flexible responding, we investigated how DMS CB1R signaling impacts local inhibitory synaptic transmission in male and female Long Evans rats. We recorded spontaneous inhibitory postsynaptic currents (sIPSC) from DMS neurons at baseline and after application of a CB1R agonist, WIN 55,212-2. We found that male rats showed decreased sIPSC frequency compared with females and that CB1R activation reduced DMS inhibitory transmission independent of sex. Altogether our results demonstrate that DMS CB1Rs regulate pavlovian devaluation sensitivity and DMS inhibitory synaptic transmission and suggest that basal sex differences in inhibitory synaptic transmission may underly sex differences in DMS function and behavioral flexibility.

## Significance Statement

Adaptive behavior requires both flexible and habitual actions depending on environmental conditions. The dorsal striatum regulates shifts from flexible to habitual behaviors, and the dorsomedial striatum (DMS) endocannabinoid (eCB) system regulates this shift in instrumental reward devaluation. Individual and sex differences in pavlovian reward devaluation suggest differences in eCB regulation of behavioral flexibility in the DMS. The current study (1) falsifies the hypothesis that DMS cannabinoid receptor-1 (CB1R) signaling promotes rigid behaviors, finding instead that DMS CB1R signaling promotes flexibility in pavlovian devaluation, and (2) establishes sex differences in pavlovian devaluation and DMS inhibitory synaptic transmission.

## Introduction

Impairments in behavioral flexibility occur across a range of mental health disorders including substance use disorder, schizophrenia, obsessive-compulsive disorder, and depression (Kalivas and Volkow, 2005; Thoma et al., 2007; Jordan and Andersen, 2017; Listunova et al., 2018; Simmler and Ozawa, 2019; Geramita et al., 2020). Preclinical studies suggest that sex and individual differences influence behavioral control when environmental conditions change from what is expected (Morrison et al., 2015; Nasser et al., 2015; Amaya et al., 2020; Keefer et al., 2020; Bien and Smith, 2023). Understanding the neurobiological underpinnings of individual and sex differences in behavioral flexibility may help identify novel therapeutic targets for disorders of behavioral control.

Instrumental conditioning procedures in rodents reveal dorsal striatal (DS) regulation of behavioral flexibility, which involves dorsomedial and dorsolateral striatal (DMS, DLS) subdivisions. The shift from goal-directed to habitual behavior that occurs after extended instrumental experience is mediated by a shift from DMS to DLS control, respectively (Dickinson et al., 1995; Ragozzino et al., 2002; Yin et al., 2004, 2005; Gremel and Costa, 2013; Amaya and Smith, 2018; Peak et al., 2019). One of the most abundant receptor types in the DS is the cannabinoid receptor-1 (CB1R), which is a G-protein–coupled receptor that inhibits local synaptic transmission. CB1Rs are expressed presynaptically on glutamatergic inputs and locally on terminals of fast-spiking interneurons and GABAergic medium spiny neurons (MSNs; Gerdeman and Lovinger, 2001; Gerdeman et al., 2002; Lovinger and Mathur, 2012; Mathur et al., 2013; Wu et al., 2015). An instrumental study shows that CB1R deletion in the orbitofrontal cortex–DS projection promotes devaluation sensitivity during schedules of reinforcement that ordinarily drive habitual responding (Gremel et al., 2016), suggesting that CB1R-mediated inhibition of DS glutamatergic inputs promotes rigid, devaluation-insensitive instrumental actions.

A primary goal of this study is to determine whether DMS CB1R signaling also promotes rigid, devaluation-insensitive pavlovian behaviors. A secondary goal of this study is to evaluate individual and sex differences in DMS CB1R control of rigid and flexible behaviors. The sign-tracking and goal-tracking model uncovers considerable individual-, sex-, and experience-dependent differences in pavlovian devaluation sensitivity (Flagel et al., 2009; Pitchers et al., 2015; Madayag et al., 2017; Keefer et al., 2020; Kochli et al., 2020). After limited training (<10 sessions) in pavlovian lever autoshaping (PLA), in which an insertable lever cue predicts a food outcome, goal-tracking (GT) rats show sensitivity to outcome devaluation while sign-tracking (ST) rats do not (Morrison et al., 2015; Nasser et al., 2015; Patitucci et al., 2016; Keefer et al., 2020). After extended training (>10 sessions), both GT and ST rats show sensitivity to satiety-induced outcome devaluation (Keefer et al., 2020), an effect established in male rats. Female rats show a more lever-directed approach during PLA and are more likely to be characterized as ST rats compared with males (Hammerslag and Gulley, 2014; Pitchers et al., 2015; Madayag et al., 2017; King et al., 2020; Kochli et al., 2020; Keefer et al., 2022), suggesting they may be less sensitive to outcome devaluation even after extended training. Indeed, in some studies examining sex differences, females are less sensitive to instrumental and pavlovian devaluation (Quinn et al., 2007; Schoenberg et al., 2019; Bien and Smith, 2023; Sood and Richard, 2023). In the present study, we use the intracranial CB1R inverse agonist, rimonabant, to determine the role of DMS CB1Rs in mediating pavlovian devaluation sensitivity in male and female rats screened in pavlovian lever autoshaping to determine tracking phenotypes.

Consistent with prior studies demonstrating sex differences in devaluation sensitivity, we find that male, but not female, rats are sensitive to pavlovian outcome devaluation. Opposite to our prediction, based on the established role of DMS CB1R to promote rigid devaluation-insensitive actions in instrumental settings, we find that DMS CB1R signaling promotes a flexible devaluation-sensitive approach in pavlovian settings. Within the canonical model that DMS controls goal-directed devaluation sensitivity, our findings suggest that CB1R-mediated inhibition of GABAergic synaptic transmission in DMS could control pavlovian devaluation sensitivity in male rats. To begin investigating this possibility, we aimed to determine (1) whether there are basal sex differences in DMS GABAergic transmission that could explain sex differences in devaluation sensitivity and (2) whether male rats show CB1R-mediated inhibition of GABAergic synaptic transmission in DMS. We first recorded spontaneous inhibitory postsynaptic currents (sIPSCs) at baseline to determine whether there are existing sex differences in DMS basal inhibitory synaptic transmission. To test the endocannabinoid (eCB) regulation of DMS inhibitory synaptic transmission, we measured the effect of CB1R activation on sIPSCs in the DMS.

## Materials and Methods

### Subjects

For behavioral experiments, we used 68 Long Evans rats (33 male, 35 female; run as five cohorts) in the age range of 7–9 weeks old at the start of training for this study. All rats were double-housed upon arrival and then single-housed 24–48 h after arrival. We performed all behavioral procedures during the dark phase of the light cycle. All rats had *ad libitum* access to standard laboratory chow (Inotiv 2018 Teklad Global 18% Protein Rodent Diet; protein 24%, fat 18%, carbohydrate 58%, 3.1 kcal/g) and water before we food deprived them to maintain 90% of their baseline weight. We surgerized one cohort prior to any behavioral training and testing and surgerized the remaining cohorts after 3 d of training. There were no pre- or postsurgery differences in behavior between groups.

For slice electrophysiology experiments, we used 24 Long Evans rats (13 male, 11 female) in the age range of 9–15 weeks old at the time of slice electrophysiology recording. All rats were double-housed upon arrival. These rats had *ad libitum* access to standard laboratory chow and water before we food deprived them 24 h prior to slice electrophysiology recording.

We maintained all rats on a reverse 12 h light/dark cycle (lights off at 10:00). We performed all procedures in accordance with the “Guide for the Care and Use of Laboratory Animals” (eighth edition, 2011, US National Research Council) and with approval by the University of Maryland, Baltimore Institutional Animal Care and Use Committee.

### Apparatus

We conducted behavioral experiments in identical operant chambers (25 × 27 × 30 cm; Med Associates) located in a separate room from the animal colony. An individual sound-attenuating cubicle with a ventilation fan surrounded each chamber. One wall contained a red house light, and the opposing wall contained a food cup with photobeam detectors that rested 2 cm above the grid floor. A programmed pellet dispenser attached to the food cup and dispensed 45 mg food pellets [catalog #1811155; Test Diet Purified Rodent Tablet (5TUL); protein 20.6%, fat 12.7%, carbohydrate 66.7%, 3.44 kcal/g]. We installed one retractable lever 6 cm above the grid floor on either side of the food cup, and we counterbalanced the lever side between subjects.

### Surgical procedures

After 3 d of PLA training, we gave *ad libitum* access to food before we performed intracranial cannula placement surgery. We anesthetized 8-week-old rats with isoflurane (VetOne; 5% induction, 2–3% maintenance) and then administered the preoperative analgesic carprofen (5 mg/kg, s.c.) and lidocaine (10 mg/ml subdermal at the incision site). We placed them in a stereotaxic frame (model 900, David Kopf Instruments) over a heating pad to maintain stable body temperature throughout the surgery.

We implanted a guide cannula (23 G; Plastics One) bilaterally at an 8° angle and 1 mm above the injection site into the DMS (coordinates from bregma −0.24 mm AP, ±2.6 mm ML, and −4.5 mm DV). We determined distance from bregma using the Paxinos and Watson rat brain atlas (Paxinos and Watson, 2006). The cannula was secured to the skull with jeweler's screws and dental cement. At the end of the surgery, we inserted a dummy cannula into the guide cannula, which we only removed during infusion habituation and infusion test procedures. We moved the rats to a recovery cage over a heating pad and administered carprofen analgesic at 24, 48, and 72 h postsurgery. We gave animals 1 week of recovery before resuming behavioral procedures.

### Pavlovian lever autoshaping training

Prior to training, we exposed all rats to the food pellets in their home cage to reduce novelty to the food. Then we trained them in daily PLA sessions which lasted ∼26 min and included 25 trials of noncontingent lever presentations [conditioned stimulus (CS)] and occurred on a variable time 60 s schedule (50–70 s). At the start of the session, the houselight turned on and remained on for the duration of the session. Each trial consisted of a 10 s lever presentation and retraction of the lever followed immediately by delivery of two 45 mg food pellets into the food cup. At the end of the session, we returned the rats to their cage and colony room. We trained the rats in PLA first for 5 d to determine their tracking group and then continued training following PLA testing for a total of 12 sessions.

#### pavlovian lever autoshaping testing

We tested the effects of blocking DMS CB1R during reinforced PLA sessions. We infused two doses of rimonabant (1 or 2 µg/µl SR141716; dissolved in 1:1:18 ethanol–emulphor–saline solution) or vehicle bilaterally into DMS at a rate of 0.5 µl/min over the span of 1 min. Rimonabant has a high affinity for CB1Rs and exerts measurable effects on behavior at doses including 1 µg/µl and lower, as shown in prior work (Oleson et al., 2012; Wenzel and Cheer, 2018). We left the infusion cannula in place for an additional minute before slowly removing it and replacing the dummy cannula. We waited 10 min after infusion before placing rats into the behavioral chamber and running the pavlovian lever autoshaping test. We infused a subset of rats with vehicle, low (1 µg/µl) or high (2 µg/µl) dose of rimonabant across 3 d, and we counterbalanced the dose across days.

#### Satiety-induced outcome devaluation testing

After the 12th training session, we gave the rats two sessions of satiety-induced outcome devaluation. The rats had 1 h of *ad libitum* access to 30 g of either their homecage chow (3.1 kcal/g, valued condition) or food pellets used during PLA training (3.44 kcal/g, devalued condition) in a ceramic ramekin as in prior work (Keefer et al., 2020, 2022; Kochli et al., 2020; Gyawali et al., 2023). Within 15 min of the end of the satiation hour, we performed intra-DMS rimonabant infusions (1 µg/µl) as described in the previous section. We waited 10 min after the infusion before placing the rats into the behavioral chamber and running the lever autoshaping test. Tests consisted of 10 nonrewarded lever presentations on variable time 60 s schedule (50–70 s). Immediately after each test, we gave the rats a 30 min food choice test in their homecage which included 10 g of homecage chow and 10 g of food pellets in separate ceramic ramekins to confirm satiety was specific to the outcome they had been fed before the test session. We retrained the rats on 25 reinforced trials on a separate day between devaluation probe tests.

#### Brain slice preparation for slice electrophysiology

We anesthetized the rats with isoflurane and then perfused with chilled *N*-methyl-d-glucamine (NMDG)–modified artificial cerebrospinal fluid (NMDG-aCSF, containing the following in mM: 92 NMDG, 20 HEPES, 25 glucose, 30 NaHCO_3_, 1.3 NaH_2_PO_4_, 2.5 KCl, 5 sodium ascorbate, 3 sodium pyruvate, 2 thiourea, 10 MgSO_4_, 0.5 CaCl_2_) that had been bubbled with carbogen (95% oxygen, 5% carbon dioxide). We collected coronal sections from the DMS (350 µM) while the brain was mounted on the cutting stage and submerged in chilled, carbogen-bubbled NMDG-aCSF, using a Leica VT1200 Vibratome. We incubated slices in carbogen-bubbled, 40° NMDG solution for 5–8 min and then transferred the slices to room temperature, carbogen-bubbled HEPES holding solution (containing the following in mM: 92 NaCl, 20 HEPES, 25 glucose, 30 NaHCO_3_, 1.3 NaH_2_PO_4_, 2.5 KCl, 5 sodium ascorbate, 3 sodium pyruvate, 2 thiourea, 1 MgSO_4_, 2 CaCl_2_). We waited 1 h before making the first recordings. Sections remained in the holding solution until electrophysiological recordings were performed.

#### Recordings and bath application of drug

We visualized cells in the DMS using an Olympus BX50 light microscope. We recorded spontaneous IPSCs (sIPSC) using borosilicate, fire-polished glass pipettes with resistance in the 3–5 MΩ range. We pulled pipettes with a Narishige PC-100 pipette puller and filled them with a CsCl-based internal solution (containing the following in mM: 150 CsCl, 10 HEPES, 2 MgCl_2_*H_2_O, 0.3 Na-GTP, 3 Mg-ATP, 0.2 BAPTA). We recorded from hemisected slices that were constantly perfused with 37° carbogen-bubbled artificial cerebrospinal fluid (aCSF; containing the following in mM: 126 NaCl, 25 NaHCO_3_, 11 glucose, 1.2 MgCl_2_*H_2_O, 1.4 NaH_2_PO_4_, 2.5 KCl, 2.4 CaCl_2_), containing blockers of AMPA (DNQX, 20 µM) and NMDA (AP5, 50 µM). We perfused the recording chamber with a basic Longer Pump BT100-2J peristaltic pump. We also recorded slices submerged in a bath containing DMSO (0.065%) and 2-hydroxypropyl-beta-cyclodextrin (0.006%). We clamped cells at −60 mV using a Molecular Devices MultiClamp 700B amplifier and digitized recordings with a Molecular Devices Axon Digidata 1550B digitizer. We used Molecular Devices Clampex 10.7 software for data acquisition. We excluded recordings when the sIPSC baseline was below −200 pA, series resistance was >40 MΩ, or series resistance changed to >20% throughout the course of the experiment.

#### Measurements

For training and devaluation probe tests, we recorded the number and duration of food cup and lever contacts, the latency to contact, and the probability during the 10 s CS (lever) period. On trials with no contacts, a latency of 10 s was recorded. To determine the tracking group, we used a pavlovian conditioned approach (PavCA) analysis (Meyer et al., 2012) which quantifies behavior along a continuum where +1.00 indicates behavior is primarily lever directed (sign-tracking) and −1.00 indicates behavior is primarily food cup directed (goal-tracking). PavCA scores are the average of three separate scores: the preference score (lever contacts minus food cup contacts divided by the sum of these measures), the latency score [time to contact food cup minus the time to contact lever divided by 10 s (duration of the cue)], and the probability score (probability to make a lever contact minus the probability to make a food cup contact across the session). We used the PavCA score from the fifth day of training to determine an individual's tracking group as follows: ST rats had a PavCA score from +0.33 to +1.00, GT rats had a PavCA score from −1.00 to −0.33, and intermediates (INT) had scores ranging from −0.32 to +0.32. Rats in goal- and intermediate-tracking groups were combined into a single GT/INT group as they showed similar patterns of outcome devaluation in other studies (Nasser et al., 2015; Keefer et al., 2020). On Day 6, we were unable to record latency data for six rats and only retained lever and food cup contacts for these rats. A preference score was used in place of PavCA for rats on this day.

For devaluation probe tests, we also report the total approach (the sum of food cup and lever contacts during the 10 s CS period) and individual contact measurements. We recorded consumption on the test days and calculated the amount of pellet or chow consumed in grams during the satiety hour and during the 30 min choice test.

We processed sIPSC traces using the template search function in Molecular Devices Clampfit 10.7 software to determine event peak amplitude and event peak start time. We report these measurements in each experiment: amplitude, calculated as the peak amplitude of an event and averaged across each recording; frequency, calculated as the number of events per recording divided by the duration of the recording in seconds; and interevent interval (IEI), calculated as the inverse of the time (in seconds) between the peak of an event and the peak of the event prior and represented through a cumulative frequency distribution.

#### Histology

At the end of behavioral experiments, we deeply anesthetized rats with isoflurane and transcardially perfused 100 ml of 0.1 M sodium phosphate buffer (PBS), followed by 200 ml of 4% paraformaldehyde (PFA) in PBS. We removed brains and postfixed them in 4% PFA over night before we transferred them to 30% sucrose in dH_2_O for 48–72 h at 4°C. We rapidly froze brains in dry ice before storing them at −20°C until slicing. We sliced brains with the Leica Microsystems 1850 cryostat to collect 40 µm coronal sections in three series through the cannula placements in the DMS. We mounted sections onto gel-coated slides and then stained them with cresyl violet before coverslipping with Permount. We examined placements under a light microscope for confirmation of cannula placement in the DMS (Fig. 2*B*). We excluded 11 rats due to cannula placements being outside the region of interest.

#### Experimental design and statistical analysis

We analyzed behavioral data using SPSS 29.0 statistical software (IBM) or Prism (Graphpad Software) with mixed-design repeated-measures analysis of variance (ANOVA) or paired *t* tests, where applicable. Significant main and interaction effects (*p* < 0.05) were followed by post hoc repeated-measures ANOVA and/or Bonferroni’s correction. We used Bonferroni’s correction when performing multiple statistical tests simultaneously on a subset of appropriate pairwise comparisons (i.e., comparing responding in valued vs devalued within each group, but not comparing devalued responding between groups). Analyses included between-subject factors of tracking (ST, GT/INT), sex (male, female), and treatment (vehicle, rimonabant) and within-subject factors of session (1–12), outcome value (valued, devalued), or outcome (nonsated, sated).

For slice electrophysiology experiments, data are represented as mean ± standard error or presented as cumulative frequency distribution plots. We performed independent samples student's *t* test, two sample Kolmogorov–Smirnov tests, or Kruskal–Wallis tests with Dunn's post hoc comparisons as appropriate using either SPSS or Prism. We analyzed the mean amplitude and mean frequency data using independent samples *t* tests between males and females. We analyzed the cumulative frequency distribution of IEI between males and females using a Kolmogorov–Smirnov test and reported the effect size using Hedges’ *g*. We analyzed the cumulative frequency of IEI between DMSO and WIN conditions in the bath and between males and females using the Kruskal–Wallis test with Dunn's post hoc comparisons. The analysis included within-subject variable of bath (pre-WIN, post-WIN) and between-subject variable of sex (male, female). We removed two data points, one from each sex, based on results from Grubb's test for outliers.

## Results

### Acquisition of pavlovian lever autoshaping differs due to tracking and sex

**Figure 1.**
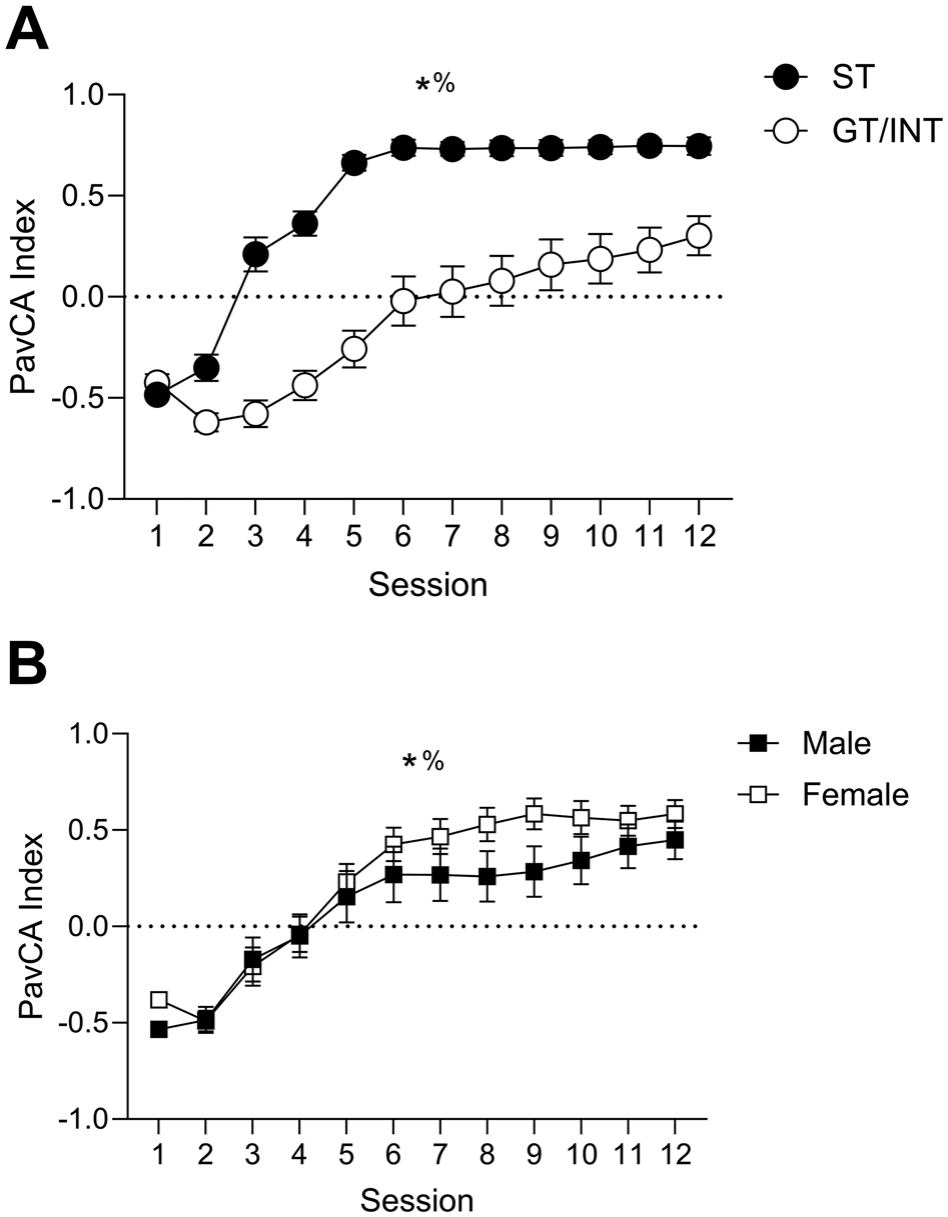
Acquisition of a pavlovian conditioned approach differs by tracking and sex. ***A***, PavCA index (mean ± SEM) split by ST and GT/INT groups (collapsed on sex). *Main effect of session. %Significant session × tracking interaction. ***B***, PavCA index (mean ± SEM) split by male and female rats (collapsed on tracking). *Main effect of session. %Significant session × sex interaction.

We trained rats for 12 d in PLA in which an insertable lever cue predicts food pellet delivery. As is standard for analyzing PLA data (Meyer et al., 2012), we used the pavlovian conditioned approach index (PavCA; Fig. 1) on the fifth session of training to determine tracking groups. Consistent with group assignments, ST rats showed more lever-directed behavior than GT/INT rats (main effect tracking; *F*_(1,53)_ = 49.293, *p* ≤ 0.001). Consistent with prior studies (Villaruel and Chaudhri, 2016; Bacharach et al., 2018; Keefer et al., 2020) showing that GT and intermediate (GT/INT) rats shift away from the food cup approach and toward the lever approach with extended training, we observed a main effect of session (*F*_(11,583)_ = 106.292, *p* < 0.001) and a session × tracking (ST, GT/INT) interaction, *F*_(11,583)_ = 13.909, *p* < 0.001 (Fig. 1*A*). Next, we examined whether there were sex differences in the acquisition and expression of pavlovian approach behaviors (Fig. 1*B*). We found a session × sex interaction for PavCA index, *F*_(11,605)_ = 1.823, *p* = 0.047). While males and females showed similar PavCA indices during initial acquisition, female rats showed more sign-tracking, via a higher PavCA index, than males with extended training (Day 8, *t*_(55) _= −1.754, *p* = 0.043; Day 9, *t*_(55) _= −2.007, *p* = 0.025). However, there were no sex differences in responding on the last day of training (PavCA index; *t*_(55) _= −1.099, *p* = 0.277), prior to testing in outcome devaluation.

### Intra-DMS inhibition of CB1R signaling impairs satiety-induced pavlovian outcome devaluation in male rats

**Figure 2.**
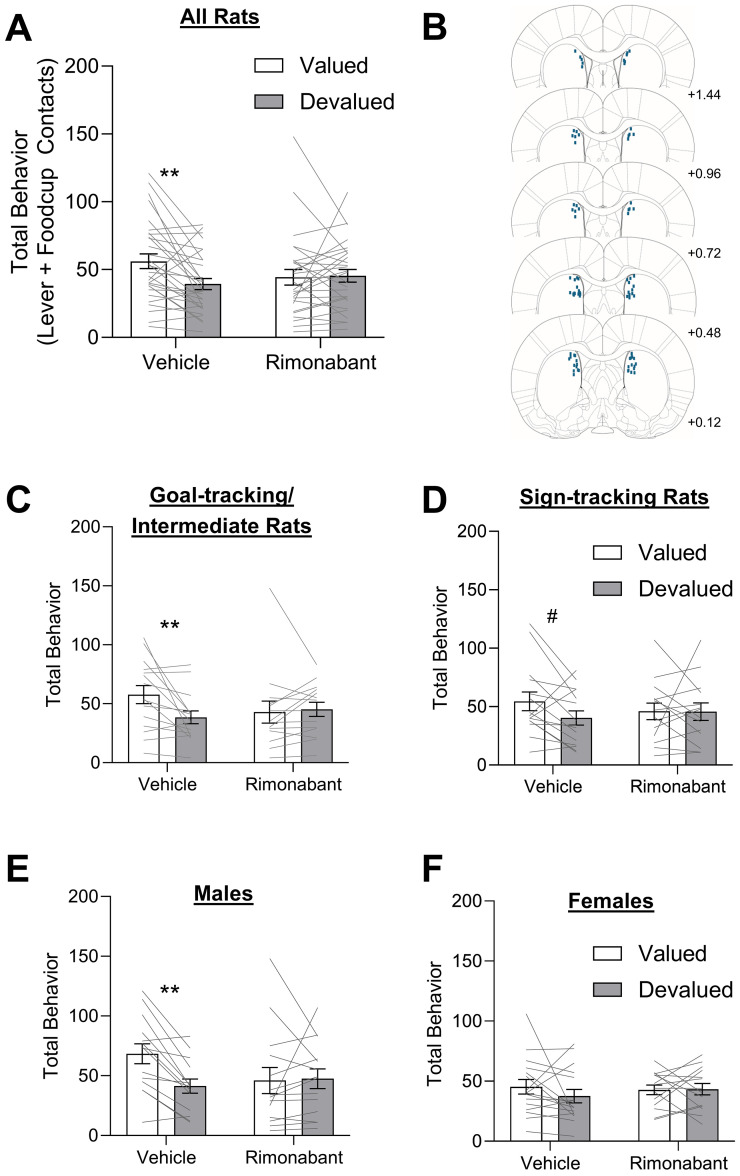
Intra-DMS CB1R signaling inhibition impairs pavlovian devaluation sensitivity, which differs by sex. Data represented as within-subject individual data (lines) and group data (bars; mean ± SEM). Rats received intra-DMS injections of either vehicle (left) or rimonabant (right) 10 min prior to the probe test. ***A***, All rats’ total behavior (sum of lever and food cup contacts) in outcome devaluation probe test. Under vehicle conditions, the rats show lower responding for devalued relative to valued conditions, and this is blocked by intra-DMS rimonabant infusions (significant outcome value × treatment interaction). ***B***, Coronal sections (in millimeters relative to bregma) depicting the location of DMS injector tips for intracranial infusions. ***C***, GT/INT rats’ total behavior in the outcome devaluation probe test is lower for devalued relative to valued conditions, and this is blocked by intra-DMS rimonabant infusions (significant outcome value × treatment interaction). ***D***, ST rats’ total behavior in outcome devaluation probe test, during which there were no significant main effects or interactions when collapsed across sex. ***E***, Male rats’ total behavior in the outcome devaluation probe test is lower for devalued relative to valued conditions, and this is blocked by intra-DMS rimonabant infusions (significant outcome value × treatment interaction). ***F***, Female rats’ total behavior in outcome devaluation probe test, during which there were no significant main effects or interactions when collapsed across tracking. Post hoc comparisons: ^#^*p* = 0.051, **p < 0.025.

We tested rats using a within-subject satiety-induced outcome devaluation procedure in which they were sated on the training pellet (devalued) or homecage chow (valued) just prior to brief PLA test sessions under extinction conditions. Prior to test sessions, we gave intra-DMS vehicle or CB1R inverse agonist, rimonabant, injections (1 µg/µl) to determine the effects of inhibiting DMS CB1R signaling on devaluation sensitivity of pavlovian approach. First, to examine whether DMS CB1R signaling inhibition affects devaluation sensitivity consistent with prior studies (Navarro et al., 2001; Hilário et al., 2007; Gremel et al., 2016), we analyzed pavlovian approach behavior in all rats as, previously, individual and sex differences were not considered. We report the total approach which is the sum of lever and foodcup contacts during the 10 s cue presentation. We compared responding during the valued (chow sated) versus devalued (pellet sated) conditions using a mixed-design repeated-measures ANOVA with between-subject factor of treatment (vehicle, rimonabant) and within-subject factor of outcome value (valued, devalued). Figure 2*A* shows the performance of all rats that received either intra-DMS vehicle or rimonabant infusions (Fig. 2*B*) during the outcome devaluation probe test. We found a main effect of outcome value (*F*_(1,56)_ = 5.558, *p* = 0.022) and an outcome value × treatment interaction (*F*_(1,55)_ = 6.663, *p* = 0.013). Under vehicle conditions, rats decreased total approach when sated on the training pellet (devalued state) compared with when they were sated on the homecage chow (valued state; *t*_(29)_ = 3.532, *p* = 0.003). In contrast, with intra-DMS rimonabant infusions, rats showed a similar amount of pavlovian approach in the valued and devalued states (*t*_(26) _= −0.086, *p* = 0.932). These results suggest a divergent CB1R-mediated mechanism for regulating pavlovian outcome devaluation in which DMS CB1R signaling promotes flexibility, in contrast to prior studies showing that DMS CB1R signaling promotes rigid responding in instrumental settings (Navarro et al., 2001; Hilário et al., 2007; Gremel et al., 2016). While these collapsed results are important to illustrate the contrasting effect of DMS CB1R signaling inhibition on pavlovian devaluation, the overall analysis ignores the potentially important factors of tracking and sex that the present study was designed to address.

Prior studies establish greater devaluation sensitivity in GT/INT than ST rats and greater devaluation sensitivity in males than females (Quinn et al., 2007; Morrison et al., 2015; Nasser et al., 2015; Patitucci et al., 2016; Smedley and Smith, 2018; Schoenberg et al., 2019; Keefer et al., 2020; Bien and Smith, 2023; Sood and Richard, 2023). Consistently, we observed an outcome value × treatment × sex × tracking interaction (*F*_(1,49)_ = 4.545, *p* = 0.038) which points to differences in the effects of treatment on devaluation sensitivity that differ by sex and/or tracking. Given previously established devaluation differences between tracking groups (Morrison et al., 2015; Nasser et al., 2015; Patitucci et al., 2016; Smedley and Smith, 2018; Keefer et al., 2020), we first examined treatment effects on devaluation sensitivity within each tracking group. In GT/INT rats, we observed a significant outcome value × treatment interaction (Fig. 2*C*, *F*_(1,27)_ = 5.377, *p* = 0.028), with greater responding in valued than devalued test under vehicle (*t*_(14)_ = 2.784, *p* = 0.0293), but not rimonabant (*t*_(13) _= −0.372, *p* = 0.716) treatments. In ST rats, we did not observe any significant main effects or interactions (Fig. 2*D*, all *F*s_(1,26)_ < 2.12, *p*s > 0.157); however, there was a trend for devaluation effect under vehicle (*t*_(14)_ = 2.136, *p* = 0.051), but not rimonabant (*t*_(12)_ = 0.176, *p* 0.863) treatments. Given previously established devaluation differences between sexes (Quinn et al., 2007; Schoenberg et al., 2019; Bien and Smith, 2023; Sood and Richard, 2023), we next examined the treatment effects on devaluation sensitivity within each sex. In male rats, we observed a main effect of outcome value and an outcome value × treatment interaction (Fig. 2*E*; value, *F*_(1,25)_ = 6.084, *p* = 0.021; value × treatment, *F*_(1,25)_ = 6.440, *p* = 0.018). Bonferroni’s post hoc comparisons confirmed that under vehicle conditions, male rats were sensitive to outcome devaluation (*t*_(13)_ = 4.670, *p* < 0.0008) responding more to the cue in valued than in devalued conditions. We observed that intra-DMS rimonabant impaired devaluation sensitivity in male rats, as they responded similarly between valued and devalued conditions (*t*_(12) _= −0.041, *p* = 0.968). In female rats, we did not observe any significant main effects or interactions (Fig. 2*F*; *F*s_(1,28) _< 0.893, *p*s > 0.353), indicating they were not sensitive to pavlovian outcome devaluation; thus, we could not evaluate treatment effects on this behavior. These analyses indicate that sex is an important factor to include as we examine treatment effects within each tracking group.

**Figure 3.**
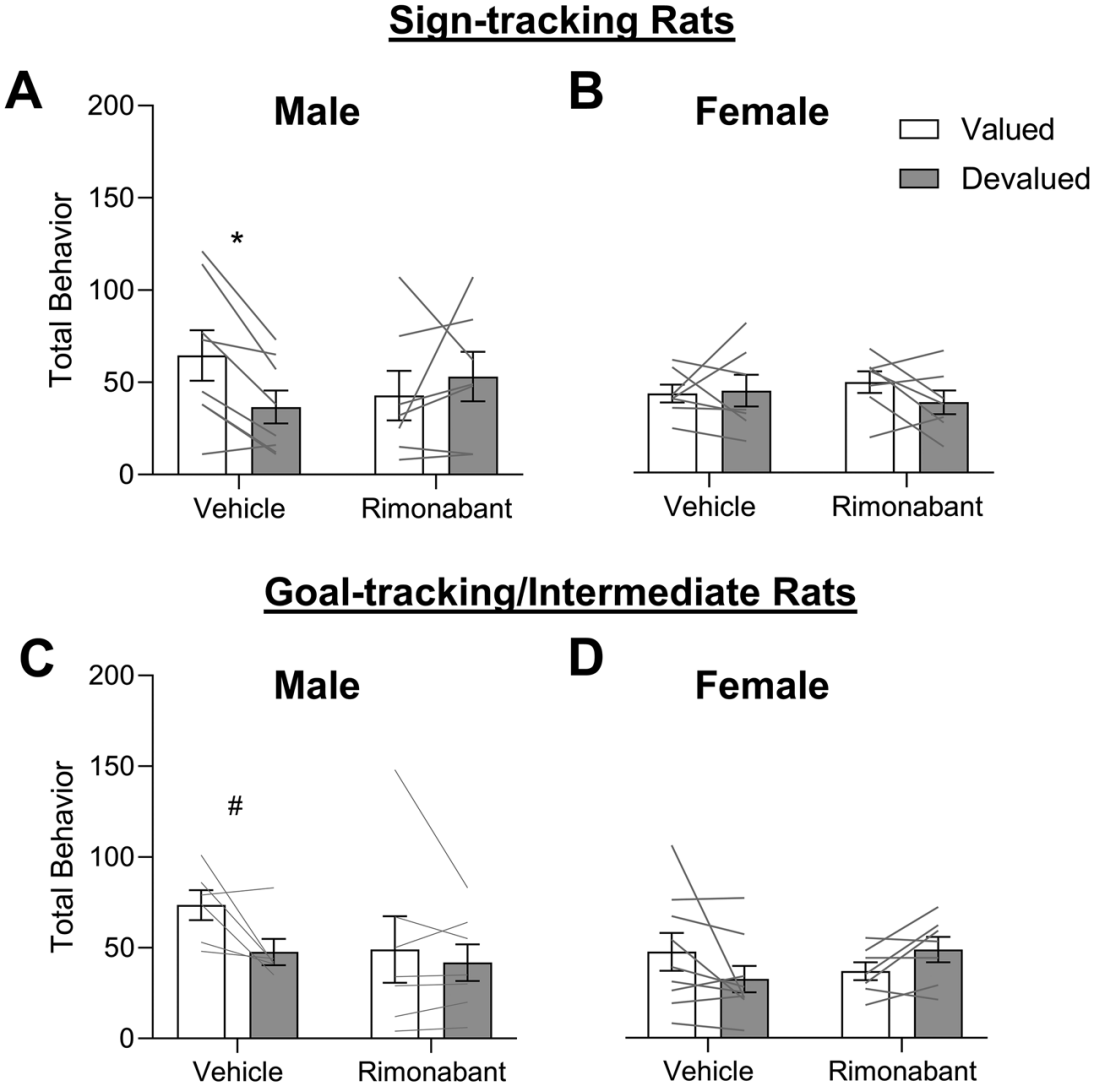
Male, but not female, rats are sensitive to pavlovian outcome devaluation, and this sensitivity is blocked by intra-DMS rimonabant regardless of the tracking group. Data represented as within-subject individual data (lines) and group data (bars; mean ± SEM). ***A***, ***B***, In ST rats, we observe a significant outcome value × treatment × sex interaction on total behavior. In male ST rats, we observed a significant outcome value × treatment interaction. Post hocs confirm that male ST rats were sensitive to devaluation with lower responding for devalued relative to valued conditions, while all other post hocs were not significant. ***C***, ***D***, In GT/INT rats, we observed an outcome value × treatment interaction, but no interaction with sex. Post hoc comparisons: ^#^*p* = 0.055, **p < 0.025. See Extended Data Figure 3-1 for an analysis of lever contacts and Extended Data Figure 3-2 for an analysis of food cup contacts.

Consistent with this conclusion, we observe an outcome value × treatment × sex interaction in ST rats (*F*_(1,24)_ = 6.210, *p* = 0.020). Furthermore, we confirmed the outcome value × treatment interaction that was observed overall (Fig. 2*A*) was also observed in male ST rats (Fig. 3*A*, *F*_(1,12)_ = 5.063, *p* = 0.044). While potentially underpowered within tracking/sex groups, post hoc analyses confirmed that under vehicle conditions, male ST rats were sensitive to devaluation (*t*_(7)_ = 3.910, *p* = 0.006, Cohen's *D* = 1.38), while intra-DMS rimonabant injections impaired devaluation sensitivity with similar levels of pavlovian approach for valued and devalued conditions (*t*_(5) _= −0.556, *p* = 0.602, Cohen's *D* = −0.26). We found similar patterns for ST rats for lever contacts (the dominant response of ST rats) during outcome devaluation (Extended Data Fig. 3-1*A*), for which there was a significant outcome value × treatment × sex interaction (*F*_(1,24)_ = 4.793, *p* = 0.039). Post hoc tests, while likely underpowered, confirmed that under vehicle conditions, male ST rats were sensitive to devaluation for lever contacts (*t*_(7)_ = 3.672, *p* = 0.032, Cohen's *D* = 1.30), while all other comparisons did not reach significance (*t*’s < 1.4, *p* > 0.200, Cohen's *D* < 0.54). As expected, due to low levels of foodcup responding, we observed no effects when analyzing male ST foodcup contacts (Extended Data Fig. 3-2*A*). In contrast to males, female ST rats showed similar levels of responding in all probe tests, and intra-DMS rimonabant had no effects (Fig. 3*B*, *F*s < 1.236, *p*s > 0.288, *t*’s < 1.5, Cohen's *D*’s < 0.58; lever, Extended Data Fig. 3-1*B*; food cup, Extended Data Fig. 3-2*B*). Notably, null effects should be interpreted with caution due to low sample sizes within tracking/sex groups.

10.1523/ENEURO.0341-24.2024.f3-1 Figure 3-1 Male ST rats are sensitive to Pavlovian outcome devaluation for lever contacts, which is blocked by intra-DMS Rimonabant*.* Data represented as within-subject individual data (lines) and group data (bars; mean ± SEM). Across all rats, we observed a significant Outcome Value X Treatment X Tracking X Sex interaction for lever contact. *A,B,* In ST rats, we observe a significant Outcome Value X Treatment X Sex interaction on lever contact*.* Post hocs confirm that male ST rats were sensitive to devaluation with lower lever contacts for devalued relative to valued conditions, while all other post hocs were not significant. *C,D* In GT/INT rats, we did not observe any significant main effects or interactions. Post hoc comparisons: *p < 0.05. See Figure 3 for analysis of total approach. Download Figure 3-1, TIF file.

10.1523/ENEURO.0341-24.2024.f3-2 Figure 3-2 Male GT/INT rats are sensitive to Pavlovian outcome devaluation for food cup responding, which is differentially affected by intra-DMS Treatment*.* Data represented as within-subject individual data (lines) and group data (bars; mean ± SEM). For all rats, we observed a significant Outcome Value X Treatment interaction and main effects of Sex and Tracking. *A, B,* In ST rats, we did not observe any significant main effects or interactions. *C, D* In GT/INT rats, we observe an Outcome Value X Treatment interaction, and a main effect of Sex. *C,* In male GT/INT rats, we observed a significant Outcome Value X Treatment interaction (%p = 0.021) but post hocs were not significant. *D,* In female GT/INTs, we did not observe any significant main effects or interactions. See Figure 3 for analysis of total approach. Download Figure 3-2, TIF file.

Consistent with prior studies, male GT/INT rats were sensitive to outcome devaluation after extended training [main effect of outcome value (Fig. 3*C*; *F*_(1,11)_ = 5.203, *p* = 0.043)], but this did not interact with treatment or sex. For GT/INT, the dominant response is food cup contacts, and for this measure, there was a significant outcome value × treatment × sex interaction (Extended Data Fig. 3-2*C,D*; *F*_(1,11)_ = 7.247, *p* = 0.012) and for males, an outcome value × treatment interaction (Extended Data Fig. 3-2*C*; *F*_(1,11)_ = 7.279, *p* = 0.021). While underpowered, post hoc tests did not reach significance for male GT/INT (vehicle, valued vs devalued *t*_(5)_ = 2.571, *p* = 0.078, Cohen's *D* = 0.86; rimonabant, valued vs devalued *t*_(6)_ = 2.4476, *p* = 0.23, Cohen's *D* = −0.27) and female GT/INT showed very little food cup behavior Extended Data Figure 3-2*D*. We observed no significant differences when analyzing lever contacts alone (Extended Data Fig. 3-1*A*, *F*s < 3.3, *p*s > 0.081). Female GT/INT rats showed a significant outcome value × treatment interaction for total behavior (Fig. 3*D*; *F*_(1,14)_ = 5.100, *p* = 0.040) that was driven by opposite patterns of behavior for the two treatments; however, differences between value conditions did not reach significance for either treatment (vehicle, valued vs devalued, *t*_(8)_ = 1.528, *p* = 0.152, Cohen's *D* = 0.53; rimonabant, valued vs devalued, *t*_(6) _= −2.113, *p* = 0.079, Cohen's *D* = −0.80). We found a similar outcome value × treatment interaction when looking at female GT/INT lever contacts alone (Extended Data Fig. 3-1*D*; *F*_(1,14)_ = 4.953, *p* = 0.043); however, none of the post hoc analyses for these measures reached significance in female GT/INT rats (vehicle, valued vs devalued, *t*_(8)_ = 0.090, *p* = 0.179, Cohen's *D* = 0.49; rimonabant, valued vs devalued, *t*_(6) _= −2.112, *p* = 0.079, Cohen's *D* = −0.80). Altogether these data indicate that male, but not female, rats are sensitive to pavlovian outcome devaluation, and this sensitivity is blocked by intra-DMS rimonabant regardless of the tracking group.

### Intra-DMS inhibition of CB1R signaling does not affect pre- or post-test consumption or pavlovian approach during nonsated sessions that were either nonreinforced or reinforced

**Figure 4.**
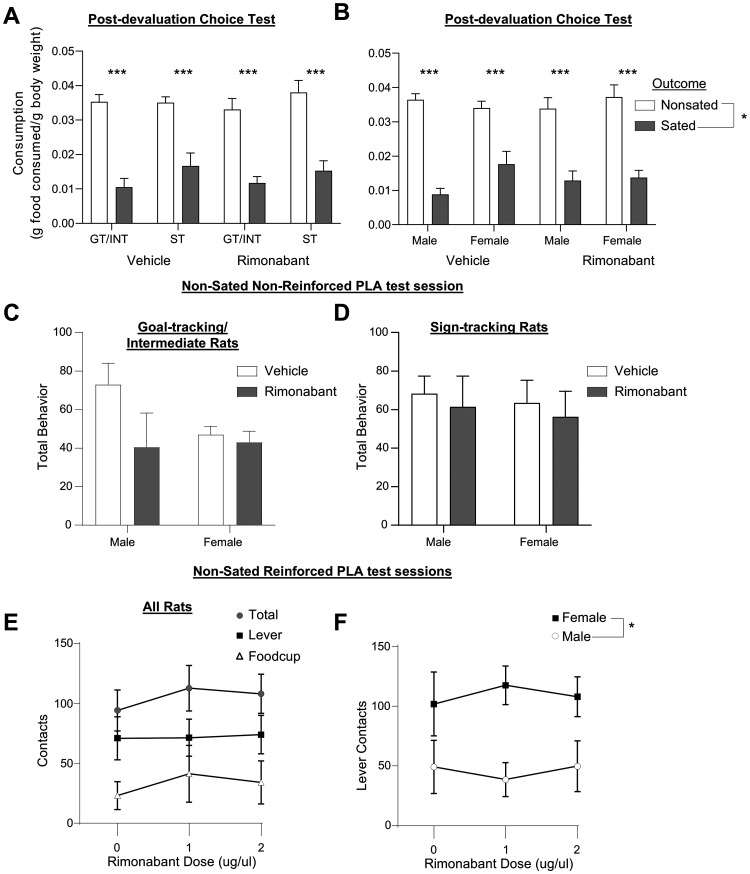
Consumption in postdevaluation choice test and conditioned responding in nonsated probe tests are unaffected by intra-DMS rimonabant. Data represented as group data (bars; mean ± SEM). ***A***, ***B***, In a post-outcome devaluation probe choice test, we gave rats 30 min of access to both outcomes, and they consumed less of the outcome they were stated on compared with nonsated outcome (*significant main effect of outcome), and there were no effects of CB1R inhibition on consumption regardless of tracking or sex. ***C***, ***D***, Intra-DMS rimonabant does not affect pavlovian conditioned approach in nonsated, nonreinforced PLA sessions. Total behavior for GT/INT rats and ST rats during a nonsated probe test identical in duration to the sated tests. There were no effects of CB1R inhibition on total behavior, regardless of tracking or sex. ***E***, ***F***, Intra-DMS rimonabant does not affect pavlovian conditioned approach in nonsated, reinforced PLA sessions. All rats received an intracranial infusion of vehicle or rimonabant (1, 2 µg/µl) 10 min prior to the start of the reinforced PLA sessions. ***E***, No effects of intra-DMS rimonabant dose on reinforced behavior for total contacts, lever, or food cup contacts and no interactions between dose or other factors. ***F***, We observed a main effect of sex on lever contacts but no effect of intra-DMS rimonabant dose. Main effect: *p = 0.05. Main effect: *p < 0.05. Post hoc comparisons: ***p < 0.01.

The observed effects of intra-DMS rimonabant on devaluation sensitivity were not due to differences in consumption between male and female rats during the 1 h satiation period. To account for body weight differences between male and female rats of the same strain and age, we normalized the amount (grams) of food consumed (either for the satiation period or postprobe choice test) to each rat's average body weight across both days of outcome devaluation tests (National Research Council, 1995; Lenglos et al., 2013). We found no difference in the amount of food consumed during the satiation period prior to the probe test (g/bw chow mean: male, 0.032, SEM ±0.002; female, 0.031, SEM ±0.002; g/bw pellet mean: male, 0.039, SEM ±0.002; female, 0.036, SEM ±0.002; *F*s < 1.153, *p*s > 0.288). To confirm the devaluation of the sated food, we gave the rats a choice test between the chow and training pellets (Fig. 4*A,B*) immediately after the end of the outcome devaluation probe test. The rats consumed less of the food that they were sated on and more of the alternative, nonsated food, verified by a main effect of outcome (*F*_(1,54)_ = 160.126, *p* < 0.001), and this did not differ by tracking (Fig. 4*A*), sex (Fig. 4*B*), or treatment (Fig. 4*A,B*, *F*s < 1.790, *p*s > 0.187).

These effects of DMS CB1R signaling inhibition were specific to the satiety-specific outcome devaluation test. In a subset of rats (*n* = 47), we gave a nonsated, nonreinforced PLA test (Fig. 4*C,D*) of the same duration (10 trials). We included between-subject factors of tracking (GT/INT, ST), sex (M, F), and treatment (vehicle, rimonabant). We found no difference in responding between intra-DMS vehicle and rimonabant groups regardless of tracking or sex (Fig. 4*C,D*; *F*s_(1,39)_ < 2.01, *p*s > 0.156). This suggests that intra-DMS rimonabant treatment effects on pavlovian approach emerge only with outcome-specific satiety.

There were also no effects of inhibiting CB1R signaling during normal PLA training sessions (nonsated, reinforced sessions). We tested the effect of intra-DMS rimonabant infusion in a subset of rats (*N* = 12, seven males, five females) during PLA training sessions identical to sessions experienced during extended training (Fig. 4*E,F*). Using a within-subject design, we found no difference between vehicle, low (1 µg/µl) or high dose (2 µg/µl) of intra-DMS rimonabant on total behavior (Fig. 4*E*; dose, *F*_(2,16)_ = 0.445, *p* = 0.648), lever presses (dose, *F*_(2,16)_ = 0.249, *p* = 0.783), or food cup contacts (dose, *F*_(2,16)_ = 0.352, *p* = 0.709) or any interactions with tracking or sex. We were underpowered to fully examine tracking and sex, but in a two-factor analysis (dose × sex) of lever contacts, we observed a main effect of sex for lever contacts (Fig. 4*F*, *F*_(1,10)_ = 5.395, *p* = 0.043), which was in line with acquisition data during which females showed more sign-tracking. Overall, rimonabant inhibition of DMS CB1R signaling did not affect the conditioned approach under reinforced conditions. Rimonabant shows a high affinity for CB1Rs and exerts measurable receptor effects at lower doses including 1 µg/µl, as shown in prior work (Wenzel and Cheer, 2018). We used the low dose (1 µg/µl) of rimonabant for outcome devaluation tests and observed behavioral effects at this dose that did not impact behavior in either reinforced or nonreinforced PLA sessions or consumption during pre- or post-tests.

Altogether, our behavioral pharmacology results suggest that CB1R signaling promotes pavlovian devaluation sensitivity, potentially via disinhibition of the DMS, via CB1R-mediated inhibition of GABAergic synaptic transmission. Within the framework that the DMS supports devaluation sensitivity, under vehicle conditions, intact CB1R signaling may be acting to reduce inhibitory synaptic transmission, promoting DMS activation and promoting flexible responding in outcome devaluation. Rimonabant infusions prevent CB1R signaling, potentially increasing inhibitory synaptic transmission onto DMS MSNs, resulting in impairments in pavlovian devaluation. Based on this, we hypothesized that DMS CB1R signaling reduces inhibitory transmission onto DMS MSNs. We reasoned that (1) basal sex differences in DMS GABAergic transmission could explain devaluation sensitivity differences between male and female rats and (2) male rats should show evidence for CB1R-mediated inhibition of GABAergic synaptic transmission in DMS.

### Baseline spontaneous IPSC recordings in DMS neurons differ between male and female Long Evans rats

**Figure 5.**
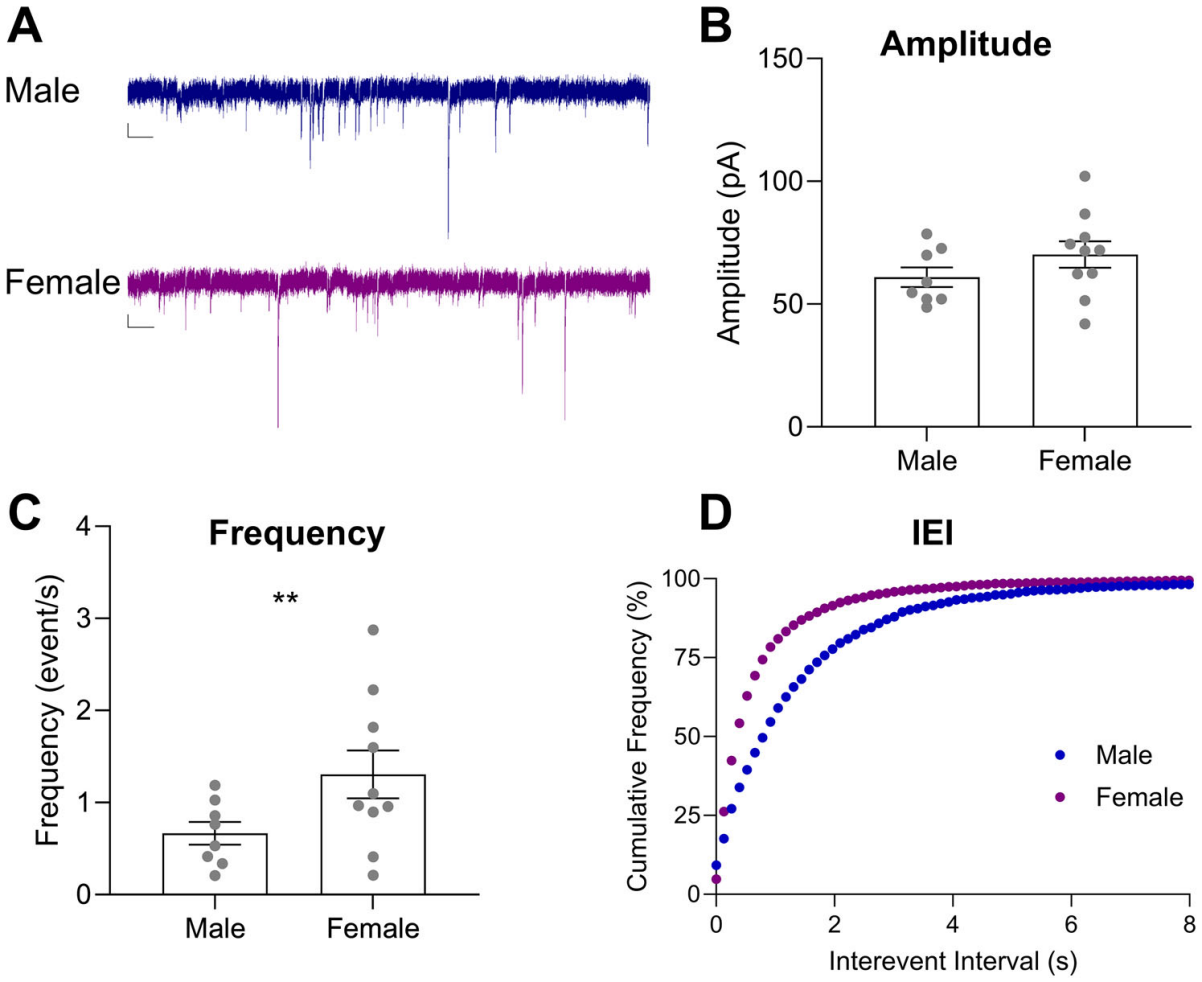
sIPSCs in DMS cells show reduced frequency and greater interevent intervals in males compared to females. ***A***, Representative sIPSC traces from DMS cells in aCSF bath from male (blue; 1–2 cells per rat; *n* = 8 cells) and female (purple; 1–2 cells per rat; *n* = 9 cells) Long Evans rats. Scale bars: 20 pA and 1 s. Data presented as mean ± SEM. ***B***, Mean amplitude. ***C***, Mean frequency. ***D***, Cumulative frequency of interevent interval of sIPSCs. **p < 0.025.

Based on the canonical model of DMS function to promote “goal-directed” devaluation sensitivity and our observation that male, but not female, rats showed pavlovian devaluation sensitivity (Fig. 2*E,F*), we predicted that male rats may show reduced inhibitory synaptic transmission in the DMS. We recorded spontaneous IPSCs from cells in the DMS in male and female rats (Fig. 5*A*, example traces). We examined the mean amplitude (absolute value), the mean frequency, or total events across the duration of the recording, and the cumulative frequency distribution for IEI, or the time between event peaks, during 5 min recordings. We found no difference in the mean amplitude of DMS sIPSCs between males and females when slices were perfused with an aCSF bath (Fig. 5*B*, *t* = −1.226, *p* = 0.239). However, we found a difference in both the frequency and IEI. Cells from male rats showed a lower frequency as compared with females (Fig. 5*C*, *t* = −2.561, *p* = 0.022) and a larger interevent interval (Fig. 5*D*, Kolmogorov–Smirnov test, *D* = 0.2498, *p* < 0.0001, Hedge's *g *= 0.426). This difference in frequency and interevent interval of sIPSCs suggests that male rats have less inhibitory synaptic transmission onto recorded DMS neurons than females.

### WIN 55,21-2 bath application changes sIPSC measures in both males and females relative to DMSO bath application

**Figure 6.**
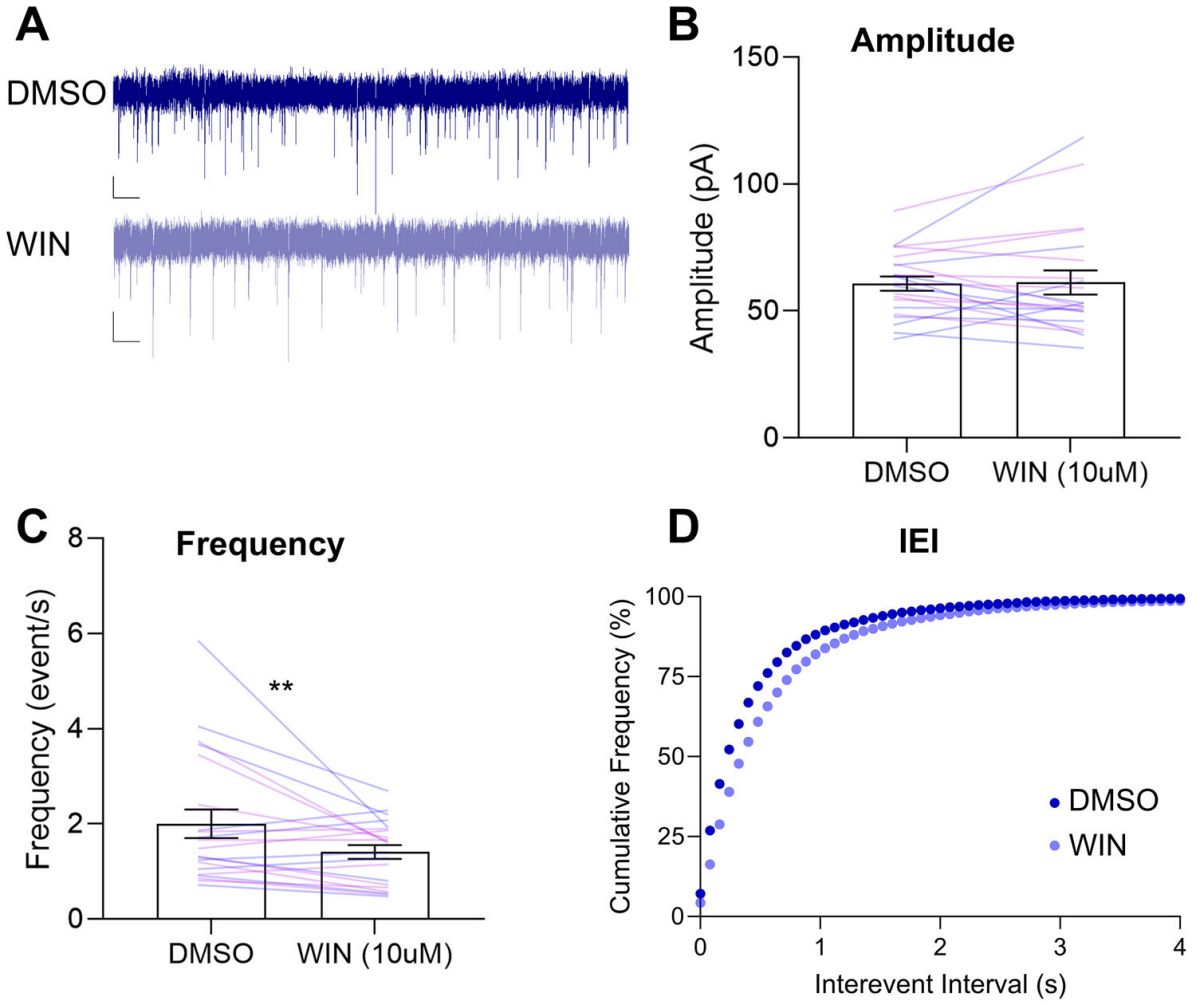
Activation of CB1R by WIN reduces sIPSC frequency and increases sIPSC interevent interval, regardless of sex. ***A***, Representative sIPSC traces from DMS cells pre- (blue) and post-WIN (light blue) bath application from Long Evans rats (1–2 cells per rat; *n* = 21 cells). Scale bars: 20 pA and 1 s. Data presented as mean ± SEM. ***B***, Mean amplitude with individual data for males (blue lines) and females (purple lines). ***C***, Mean frequency with individual data for males and females. ***D***, Cumulative frequency of interevent interval of sIPSCs. **p < 0.025.

Based on the canonical model of DMS function to promote “goal-directed” devaluation sensitivity and our observation that male rats showed pavlovian devaluation sensitivity that was blocked by CB1R inhibition (Fig. 2*E*), we predicted that male rats would show evidence for CB1R-mediated inhibition of GABAergic synaptic transmission in DMS. We hypothesized that activation of DMS CB1R would reduce inhibitory synaptic transmission in male rats and included females to investigate if there were sex differences in the effect of CB1R manipulation on sIPSCs in the DMS. We recorded sIPSCs from DMS cells for 5 min at baseline and following a 10 min bath application of a CB1R agonist, WIN 55,212-2 (WIN, 10 µM; Fig. 6*A*). We used a CB1R agonist because we were recording spontaneous activity in unstimulated DMS sections (Fig. 6*A*, example traces), and did not expect basal eCB tone to be high enough to measure the effects CB1R inhibition. eCB release occurs in an activity-dependent manner and requires depolarization of the postsynaptic cell (Di Marzo et al., 1994; Di et al., 2005; Hashimotodani et al., 2007). Despite this limitation, we aimed to determine whether male rats show CB1R-mediated inhibition of GABAergic synaptic transmission in the DMS. Using the CB1R agonist approach, we found that there were no differences in the mean amplitude of sIPSCs due to WIN or sex (Fig. 6*B*, *F*s < 1.182, *p*s > 0.290). However, we found differences in frequency and interevent interval (Fig. 6*C,D*). We found a main effect of WIN for frequency (*F*_(1,19)_ = 6.306, *p* = 0.021) but no main effect or interaction with sex (*F*s < 0.825, *p* > 0.375). Both males and females showed a lower sIPSC frequency following WIN application; however, the frequency change in males was not statistically significant when analyzed using a paired-sample *t* test (male, *t*_(9)_ = 1.603, *p* = 0.072; female, *t*_(10)_ = 2.124, *p* = 0.030). We found that application of WIN shifted the IEI cumulative distribution curves to the right (Kruskal–Wallis, *H* = 1,359, *p* < 0.001), and post hoc comparisons confirmed that this occurred for both male and female rats (DMSO vs WIN; Dunn's comparisons; male, *p* < 0.0001, Hedges’ *g *= 0.2085; female, *p* < 0.0001, Hedges’ *g* = 0.2291). This rightward shift suggests that WIN increases the IEI in both sexes. Application of WIN in the bath caused a reduction in the frequency of inhibitory events and an increase in the interevent interval across all rats, suggesting that CB1R located on presynaptic inhibitory inputs suppresses the release of GABA in the DMS.

## Discussion

In the current studies, we investigated the role of DMS CB1R signaling in pavlovian outcome devaluation and regulation of inhibitory synaptic transmission. We found that after extended training in PLA, males were sensitive to outcome devaluation, while females were not, and that DMS CB1Rs were necessary for the devaluation sensitivity in males. Slice electrophysiology studies revealed a reduced frequency of inhibitory synaptic events in DMS neurons of males as compared with female rats. However, activation of DMS CB1Rs reduced the frequency and increased the IEI of sIPSCs in both sexes.

The current results align with prior research that establishes significant individual-, experience-, and sex-dependent differences in pavlovian devaluation. Consistent with previous studies (Pitchers et al., 2015; Kochli et al., 2020; Keefer et al., 2022), we found that female rats showed more lever-directed behaviors than males during extended training, but this difference diminished before testing in outcome devaluation. Under vehicle conditions, we replicated prior findings that male rats show devaluation sensitivity after extended training in PLA (Keefer et al., 2020). We extended this work to include females, for which we did not observe devaluation sensitivity after extended training (Fig. 2). These results echo the findings of other studies that indicate females are less sensitive to instrumental and pavlovian devaluation (Quinn et al., 2007; Schoenberg et al., 2019; Bien and Smith, 2023; Sood and Richard, 2023). However, null devaluation effects for females should be interpreted with caution, particularly within specific tracking groups as we were less powered to detect effects in those analyses. It is also possible there are other behaviors (i.e., conditioned orienting and post-CS responding) that are not measured here for which females may express behavioral flexibility (Sood and Richard, 2023). Satiety procedures used *ad libitum* access to homecage chow or food pellets as in prior work (Keefer et al., 2020, 2022; Kochli et al., 2020; Gyawali et al., 2023). We observed neither a difference in pellet versus chow consumed nor any sex differences in the amount of chow or pellets consumed during the satiation period. However, satiety procedures using two foods of similar palatability and caloric density would be ideal in future investigations of sex differences in behavioral flexibility.

At first, we predicted that dorsal striatal CB1R signaling would promote rigid, or habitual, behaviors as has been shown for instrumental outcome devaluation (Gremel et al., 2016). However, our study suggests that CB1Rs in DMS promote behavioral flexibility in male rats, running counter to this established understanding. There are several factors that may have contributed to the divergence of results including species differences, the use of pavlovian versus instrumental devaluation procedures, and the subregion-specific effects of experimental manipulations. The prior study trained CB1R flox mice in both random ratio (RR) and random interval (RI) schedules of instrumental reinforcement and generated an OFC-DS–specific CB1R knock-out. Our study used Long Evans rats trained in a pavlovian task. Competing action–outcome and stimulus–response associations mediate instrumental devaluation, and studies show that goal-directed behaviors shift to habit with extended training even under RR schedules of reinforcement (Adams and Dickinson, 1981; Adams, 1982; Gremel et al., 2016). This is not the case with pavlovian behaviors that are sensitive to devaluation even after extended training (Holland, 1998; Keefer et al., 2020), suggesting that stimulus–outcome associations support adaptive reward seeking despite overtraining. Thus, differences in pavlovian and instrumental processes may, in part, underlie divergent findings between studies. Another possibility is methodological differences in the way CB1R was manipulated between studies. CB1R deletion in the OFC-DS projection promoted “goal-directed” devaluation sensitivity even during RI schedules of reinforcement that ordinarily drive “habitual” devaluation insensitivity (Gremel et al., 2016). Our current study inhibited CB1R signaling indiscriminately—likely affecting both inhibitory and excitatory synaptic transmission—rather than specifically glutamatergic OFC afferents to the dorsal striatum (DS), as in the prior study. Nevertheless, prior work shows that systemic activation of CB1Rs promotes rigid responding (Hilário et al., 2007; Nazzaro et al., 2012) and while both DLS and DMS express CB1Rs (Hohmann and Herkenham, 2000; Fusco et al., 2004; Van Waes et al., 2012), more of the CB1R work within subregions of the DS focuses on the DLS. The DLS does express CB1R more densely than DMS; thus, it is possible that off-target effects impact DLS function, an area with high CB1R density (Hohmann and Herkenham, 2000; Fusco et al., 2004; Van Waes et al., 2012), and this could confound our results. We think this is unlikely given the volume of rimonabant infusions (0.5 µl per hemisphere) and our ex vivo confirmation of reduced inhibitory synaptic transmission with CB1R activation in the DMS.

The current targeting of DMS, as compared with DLS, may in part explain why our results diverge from observations that DS CB1Rs support rigid responding via inhibition of glutamatergic inputs. Our findings fit within the context of the DMS’ role of biasing behavior toward “goal-directed” responding. Reducing the activity of the DMS through lesion or pharmacological inhibition impairs flexible responding in a variety of tasks (Yin et al., 2005; Corbit and Janak, 2010; Gremel and Costa, 2013; Li et al., 2022). These prior studies establish that the DMS supports flexible, goal-directed instrumental conditioned responding. To be interpreted in this canonical framework, our behavioral pharmacology results suggest that CB1R signaling promotes flexible responding via disinhibition of the DMS, perhaps via CB1R-mediated inhibition of GABAergic synaptic transmission. Within this framework, under vehicle conditions intact CB1R signaling reduces inhibitory synaptic transmission, promoting both DMS activation and flexible responding. Rimonabant infusions prevent CB1R signaling, increasing inhibitory synaptic transmission onto DMS MSNs, resulting in impaired “goal-directed” pavlovian devaluation sensitivity. Consistent with this framework, we found that DMS CB1R signaling reduced inhibitory transmission in DMS, which would support DMS activation and promote pavlovian devaluation sensitivity. We also found evidence for basal sex differences in GABAergic transmission that could explain devaluation sensitivity differences between male and female rats.

Our slice electrophysiology studies focused on inhibitory synaptic currents to investigate this hypothesis. At baseline, we found that males showed reduced inhibitory events as compared with females (Fig. 5). Within the above framework of striatal contributions to goal-directed and habitual control of behavior, lower levels of DMS inhibitory transmission (as seen in males) would promote flexibility, and higher levels of inhibitory transmission (as seen in females) would prevent the expression of outcome devaluation, consistent with our devaluation findings in male and female rats, respectively. While we did not confirm the identity of the cells we recorded, approximately 90% of cells across the DS are medium spiny neurons (MSNs), the main type of projection neurons arising from the striatum (Graveland and Difiglia, 1985). Due to their abundance, we likely recorded from MSNs in the DMS. Multiple studies have shown that intact female rats and males treated with estradiol have increased striatal MSN excitability (Tansey et al., 2008; Dorris et al., 2015; Cao et al., 2018; Proaño et al., 2018) and estradiol decreases GABA release (Schultz et al., 2009). However, these studies are not specific to the DMS. Additionally, some studies have shown lower numbers of GABAergic neurons in males compared with females (Ovtscharoff et al., 1992), which may explain reduced inhibitory synaptic transmission in males. However, there are many types of GABAergic cells in the DMS. GABAergic medium spiny neurons (MSNs) are the main projection neurons of the DMS, and they also project locally to other MSNs (Wilson and Groves, 1980; Somogyi et al., 1981; Graveland and Difiglia, 1985; Czubayko and Plenz, 2002; Tunstall et al., 2002; Burke et al., 2017). There are also multiple GABAergic interneuron types, predominately parvalbumin-positive fast-spiking interneurons (FSIs) and somatostatin interneurons (SOM). In fact, a study focusing on sex differences in the number of interneurons found that some GABAergic interneurons are more dense in males than females (FSIs) while other interneurons are less dense in males than females (Van Zandt et al., 2024). Thus, further work must be done to isolate inhibitory synaptic transmission from these different sources and better understand sex differences in the DMS with cell-type specificity.

We showed that CB1R activation reduced the frequency of inhibitory events regardless of sex (Fig. 6). This should be interpreted with caution, as we only tested a single dose of the CB1R agonist. We applied WIN 55,212-2 at a concentration of 10 µM, which was a high concentration for bath application. Other studies use much lower doses (1 µM) and report sex differences in other brain regions such as the hippocampus (Tabatadze et al., 2015; Ferraro et al., 2020). Both males and females express CB1R in the DS, and males express CB1R more densely in the striatum and other brain regions than females (Laurikainen et al., 2019; Liu et al., 2020). Thus, it is possible that application of WIN at a lower dose may reveal more sensitivity to CB1R manipulation in males due to this higher concentration of receptors.

Our in vivo behavioral pharmacology experiment revealed sex differences in devaluation sensitivity, in which male rats were flexible while female rats were not. Consistent with our DMS disinhibition hypothesis, we found that male rats showed reduced DMS inhibitory synaptic transmission in our ex vivo slice electrophysiology experiments. Under vehicle conditions, intact CB1R signaling reduces inhibitory synaptic transmission, potentially promoting DMS activation and flexible responding in male rats. Additionally, we find that male rats require DMS CB1R signaling to express flexible responding. In DMS slices, we saw evidence for CB1R-mediated inhibition of GABAergic synaptic transmission in male rats. While not directly tested, this suggests a viable mechanism by which rimonabant infusions block DMS disinhibition and impair “goal-directed” pavlovian devaluation sensitivity in male rats. There are many possibilities that remain untested regarding sex differences in DMS CB1R regulation of behavioral flexibility: (1) males may express more DMS CB1Rs, (2) males may have enhanced DMS CB1R function, or (3) outcome devaluation procedures may result in greater eCB release in males than females. Our slice electrophysiology studies suggest that DMS CB1R expression does not differ between males and females as both sexes are sensitive to CB1R activation, though a full dose–response for CB1R agonist would be needed to rule out this possibility. Downstream of the CB1R in other brain regions, CB1R receptor function and intracellular signaling differ between males and females (Tabatadze et al., 2015), and this may be the case in DMS. Upstream of CB1R signaling, males and females may differ in DMS eCB release dynamics, though this has yet to be investigated.

Caveats of these electrophysiological findings are that we recorded from behavior-naive rats with limited food restriction. It is possible that behavioral experience alters DMS inhibitory tone or changes DMS activity, as has been shown in other studies examining DMS activity after extended training or under different schedules of reinforcement (Fanelli et al., 2013; Gremel and Costa, 2013; Vandaele et al., 2019). Additionally, food restriction levels interact with schedules of reinforcement to control task engagement in outcome devaluation and may also influence DMS engagement and associated neurophysiology (Chevée et al., 2023). Recent studies show that even short-term food restriction alters DS physiology (Campanelli et al., 2021). While no studies to date correlate the length of food restriction with changes to DMS physiology specifically, it is possible that the limited food restriction procedure in our slice electrophysiology experiments limited the detection of sex differences or obscured other DMS physiology changes that we may have seen with longer restriction time periods used in behavioral studies.

The difference in CB1R manipulations (inverse agonist vs agonist approaches) between in vivo pharmacology and ex vivo physiology experiments limits some of our conclusions. For the in vivo behavioral pharmacology experiments, we used an inverse agonist, rimonabant, because we expected behavioral conditions to activate DMS. DMS neuron activation causes the release of both major eCBs, anandamide and 2-arachidonoylglycerol (AEA and 2-AG; Ade and Lovinger, 2007; Maccarrone et al., 2008). Thus, we expected DMS eCB tone to be high enough to inhibit CB1R signaling, via inverse agonism, and examine its effects on behavior. However, when transitioning to ex vivo slice electrophysiology in the DMS, we recorded spontaneous synaptic events without stimulation of DMS neurons, and did not expect DMS eCB release under these conditions. eCB release occurs in an activity-dependent manner and requires depolarization of the postsynaptic cell (Di Marzo et al., 1994; Di et al., 2005; Hashimotodani et al., 2007). Despite this limitation, we aimed to determine whether male rats show CB1R-mediated inhibition of GABAergic synaptic transmission in DMS. Thus, we used WIN to induce CB1R activation and found it decreased DMS inhibitory synaptic transmission in male and female rats. Both drugs bind to the same location on the receptor but exert different effects: WIN promotes activation of all CB1Rs, while rimonabant promotes inactivation of constitutively active CB1Rs. However, their functions are not direct opposites due to the difference in affinities for the CB1R (Rinaldi-Carmona et al., 1994). Rimonabant shows a higher affinity for CB1Rs than WIN and will exert measurable receptor effects at lower doses. Ideally, the effects of rimonabant ex vivo could be examined in future slice electrophysiology experiments that utilize electrical or optogenetic stimulation procedures to increase activity-dependent eCB release and allow for testing of rimonabant effects on DMS inhibitory synaptic transmission ex vivo. Incorporating DAG or MAG lipase inhibitors would elucidate eCB ligand-specific (2-AG and AEA) contributions to DMS-mediated behavioral flexibility and synaptic transmission.

CB1Rs are located on multiple cell types in the DS, so further work must be done to identify the cell type that supports pavlovian flexibility in male rats. One notable possibility is the parvalbumin-positive FSIs. CB1Rs are expressed on striatal PV-FSIs and mediate a form of inhibitory LTD that disinhibits MSNs, a mechanism that is associated with striatal regulation of behavioral flexibility (DePoy et al., 2013; Mathur et al., 2013). CB1Rs are also expressed on cortical inputs that target MSNs and MSNs themselves (Gerdeman and Lovinger, 2001; Gerdeman et al., 2002; Lovinger and Mathur, 2012; Wu et al., 2015; Lovinger et al., 2022), but it has not yet been established whether cortical projections targeting PV-FSIs also contain CB1Rs. CB1R signaling at corticostriatal FSI synapses would be expected to reduce inhibitory tone and increase DMS MSN activation, a similar result to CB1R signaling at FSI-MSN synapses. Direct manipulation of DLS PV-FSIs shows that their activity is critical to supporting habitual responding (O’Hare et al., 2017; Patton et al., 2020) but much less is known about DMS PV-FSIs and their contribution to habitual or goal-directed responding. Thus, these two hypotheses must be tested to discover the cell-type–specific mechanism of DMS CB1R regulation of pavlovian devaluation sensitivity.

Overall, the current study showed that males are sensitive to pavlovian outcome devaluation, a result that may be explained by reduced inhibitory synaptic transmission in the DMS. We found that the devaluation sensitivity of male rats requires DMS CB1R, but more work is needed to identify the cell-type specific population of CB1Rs that support flexible responding. Additionally, it is possible that DMS CB1Rs would be necessary for the devaluation sensitivity of females in cases where they respond flexibly at baseline, as in illness-induced outcome devaluation (Bien and Smith, 2023). Thus, future studies should manipulate DMS CB1Rs under conditions in which males and females respond similarly to determine if CB1Rs play a sex-specific role in mediating behavioral flexibility.

## Synthesis

Reviewing Editor: Jibran Khokhar, Western University Department of Anatomy and Cell Biology Decisions are customarily a result of the Reviewing Editor and the peer reviewers coming together and discussing their recommendations until a consensus is reached. When revisions are invited, a fact-based synthesis statement explaining their decision and outlining what is needed to prepare a revision will be listed below. The following reviewer(s) agreed to reveal their identity: Laura Corbit.

This set of studies investigates the mechanisms underlying the sensitivity of Pavlovian conditioned approach to sensory-specific outcome devaluation in male and female rats. The authors trained rats in a Pavlovian autoshaping task, where a lever predicted a food reward. Male rats exhibited sensitivity to outcome devaluation, showing reduced behavior when pre-fed the specific reward compared to a different one. Female rats did not show this sensitivity. The study also explored the role of CB1R signaling by using inverse agonism in the dorsal medial striatum, which rendered male rats insensitive to outcome devaluation. This manipulation did not affect behavior when the reward was earned during the test, suggesting a specific effect on the incorporation of outcome value changes into behavior. In a separate experiment, the authors recorded inhibitory currents in striatal medium spiny neurons, finding a sex difference in these currents and exploring the effects of CB1R agonism. While the synaptic physiology findings are interesting, they are limited by the focus on inhibitory currents and lack of direct validation of the behavioral effects observed with rimonabant.

Major Concerns

- The manuscript combines two distinct studies-a behavioral neuropharmacological investigation and synaptic physiology exploration-with little connection between the two findings, making it difficult to evaluate the evidence for either study. Clarifying the narrative and the logic behind including both studies will be helpful.

- Differences in pharmacology (inverse agonism vs. agonism) between studies are not reconciled, complicating the interpretation of results.

- The manuscript lacks a strong link between the behavioral data and synaptic physiology findings.

- The organization and presentation of results are confusing, with key findings difficult to follow. Many results are relegated to supplementary figures, making it challenging to assess methods and conclusions.

- Important variables like sex differences and tracking behaviors are collapsed, obscuring the effects. The paper should consistently emphasize these variables in analyses and figures.

- The exclusive focus on inhibitory currents is surprising given CB1R's role in glutamatergic transmission. The lack of excitatory current data limits interpretation.

- The synaptic physiology findings are not well linked to the observed behavioral outcomes.

- The lack of consistency in food types used for devaluation (pellets vs. chow) complicates interpretation of consumption data.

- Differences in food deprivation protocols between behavioral and electrophysiology experiments could affect basal cannabinoid activity and outcomes.

- The use of different doses of rimonabant is not clearly presented, raising questions about dose effects.

- Small group sizes may limit the power to detect significant effects, requiring cautious interpretation of null results.

Minor Concerns

- Awkward phrasing and tense changes throughout the manuscript decrease readability.

- The introduction suggests that the amount of conditioned responding is related to sensitivity to outcome devaluation (paraphrasing for space but the sentence - "Female rats show more sign-tracking, so they may be less sensitive"). Is this true or has this been demonstrated in this study or others?

- Abbreviations are used inconsistently, reducing accessibility for readers.

- Clarification is needed for the use of Bonferroni correction in statistical tests.

- The description of lever delivery should use "variable time" instead of "variable interval" to accurately describe the procedure.

- Sex differences in electrophysiology data should be consistently reported, even if no differences are observed with the drug.

- The manuscript should include simple effects (devaluation effects for each group) to support conclusions drawn.

- The authors should rethink what is included as extended vs. primary data.

## Author Response

We thank the reviewers for their input and the editor for the concise and useful synthesis of reviews. We address each point below in blue text, and textual changes in the manuscript appear in bold font. We revised our figures to include some of the previously supplemental findings and moved most to the main text.

Synthesis Statement for Author (Required):

This set of studies investigates the mechanisms underlying the sensitivity of Pavlovian conditioned approach to sensory-specific outcome devaluation in male and female rats. The authors trained rats in a Pavlovian autoshaping task, where a lever predicted a food reward. Male rats exhibited sensitivity to outcome devaluation, showing reduced behavior when pre-fed the specific reward compared to a different one. Female rats did not show this sensitivity. The study also explored the role of CB1R signaling by using inverse agonism in the dorsal medial striatum, which rendered male rats insensitive to outcome devaluation. This manipulation did not affect behavior when the reward was earned during the test, suggesting a specific effect on the incorporation of outcome value changes into behavior. In a separate experiment, the authors recorded inhibitory currents in striatal medium spiny neurons, finding a sex difference in these currents and exploring the effects of CB1R agonism. While the synaptic physiology findings are interesting, they are limited by the focus on inhibitory currents and lack of direct validation of the behavioral effects observed with rimonabant.

Major Concerns - The manuscript combines two distinct studies-a behavioral neuropharmacological investigation and synaptic physiology exploration-with little connection between the two findings, making it difficult to evaluate the evidence for either study. Clarifying the narrative and the logic behind including both studies will be helpful.

- The manuscript lacks a strong link between the behavioral data and synaptic physiology findings.

We added additional rationale for combining these experiments throughout the manuscript.

Intro text (line 106): "Consistent with prior studies demonstrating sex differences in devaluation sensitivity, we find that male, but not female, rats are sensitive to Pavlovian outcome devaluation. Opposite to our prediction based on the canonical model of DMS activation to promote devaluation sensitivity, we find that DMS CB1R signaling, which inhibits synaptic transmission, promotes Pavlovian outcome devaluation sensitivity in male rats. This runs counter to the role for CB1R-mediated inhibition of glutamatergic synaptic transmission in DMS in promoting rigid, devaluation-insensitive instrumental actions. Within the framework of the canonical model of DMS function to promote devaluation sensitivity, our findings instead suggest CB1R-mediated inhibition of GABAergic synaptic transmission in DMS promotes Pavlovian devaluation sensitivity in male rats. To begin investigating this possibility we aimed to determine 1) whether there are basal sex differences in DMS GABAergic transmission in male and female rats that could explain devaluation sensitivity differences and 2) whether male rats show CB1R-mediated inhibition of GABAergic synaptic transmission in DMS." Results text (line 402): "Altogether, our behavioral pharmacology results suggest that CB1R signaling promotes Pavlovian devaluation sensitivity, potentially via disinhibition of the DMS via CB1R-mediated inhibition of GABAergic synaptic transmission. Within the framework that the DMS supports devaluation sensitivity, under vehicle conditions intact CB1R signaling may be acting to reduce inhibitory synaptic transmission, promoting DMS activation and promoting flexible responding in outcome devaluation. Rimonabant infusions prevent CB1R signaling, potentially increasing inhibitory synaptic transmission onto DMS MSNs, resulting in impairments in Pavlovian devaluation. Based on this, we hypothesized that DMS CB1R signaling reduces inhibitory transmission onto DMS MSNs. We reasoned that 1) basal sex differences in DMS GABAergic transmission could explain devaluation sensitivity differences between male and female rats and 2) male rats should show evidence for CB1R-mediated inhibition of GABAergic synaptic transmission in DMS." Results text (lines 414 and 428): "Based on the canonical model of DMS function to promote "goal-directed" devaluation sensitivity and our observation that male rats showed Pavlovian devaluation sensitivity that was blocked by CB1R inhibition (Fig. 2E), we predicted that..." Discussion text (line 505): "To be interpreted in this canonical framework, our behavioral pharmacology results suggest that CB1R signaling promotes flexible responding via disinhibition of the DMS, perhaps via CB1R-mediated inhibition of GABAergic synaptic transmission. Within this framework, under vehicle conditions intact CB1R signaling reduces inhibitory synaptic transmission, promoting both DMS activation and flexible responding. Rimonabant infusions prevent CB1R signaling, increasing inhibitory synaptic transmission onto DMS MSNs, resulting in impaired "goal-directed" Pavlovian devaluation sensitivity. Consistent with this framework, we found that DMS CB1R signaling reduces inhibitory transmission in DMS, which would support DMS activation and promote Pavlovian devaluation sensitivity. We also found evidence for basal sex differences in GABAergic transmission that could explain devaluation sensitivity differences between male and female rats." - Differences in pharmacology (inverse agonism vs. agonism) between studies are not reconciled, complicating the interpretation of results.

We added additional rationale for use of rimonabant for pharmacology and WIN for electrophysiology within the results and discussion sections.

Results text (line 435): "We used a CB1R agonist because we were recording spontaneous activity in unstimulated DMS sections (example traces Fig. 6A), and did not expect basal eCB tone to be high enough to measure the effects CB1R inhibition. eCB release occurs in an activity-dependent manner and requires depolarization of the post-synaptic cell (Di Marzo et al., 1994; Di et al., 2005; Hashimotodani et al., 2007). Despite this limitation, we aimed to determine whether male rats show CB1R-mediated inhibition of GABAergic synaptic transmission in the DMS. Using the CB1R agonist approach, we found that..." Discussion text (line 576): "The difference in CB1R manipulations (inverse agonist vs. agonist approaches) between in vivo pharmacology and ex vivo physiology experiments limits some of our conclusions. For the in vivo behavioral pharmacology experiments, we used inverse agonist, rimonabant, because we expected behavioral conditions to activate DMS. DMS neuron activation causes the release of both major eCBs, anandamide and 2-arachidonoylglycerol (AEA and 2-AG; Ade &Lovinger, 2007; Maccarrone et al., 2008). Thus, we expected DMS eCB tone to be high enough to inhibit CB1R signaling, via inverse agonism, and examine effects on behavior. However, when transitioning to ex vivo slice electrophysiology in the DMS, we recorded spontaneous synaptic events without stimulation of DMS neurons, and did not expect DMS eCB release under these conditions. eCB release occurs in an activity-dependent manner and requires depolarization of the post-synaptic cell (Di Marzo et al., 1994; Di et al., 2005; Hashimotodani et al., 2007). Despite this limitation, we aimed to determine whether male rats show CB1R-mediated inhibition of GABAergic synaptic transmission in DMS. Thus, we used WIN to induce CB1R activation and found it decreased DMS inhibitory synaptic transmission in male and female rats. Both drugs bind to the same location on the receptor but exert different effects: WIN promotes activation of all CB1R, while rimonabant promotes inactivation of constitutively active CB1Rs. However, their functions are not direct opposites due to the difference in affinities for the CB1R (Rinaldi-Carmona et al., 1994). Rimonabant shows a higher affinity for CB1Rs than WIN and will exert measurable receptor effects at lower doses. Ideally the effects of rimonabant ex vivo could be examined in future slice electrophysiology experiments that utilize electrical or optogenetic stimulation procedures to increase activity-dependent eCB release and allow for testing of rimonabant effects on DMS inhibitory synaptic transmission ex vivo. Incorporating DAG- or MAG- lipase inhibitors would elucidate eCB ligand specific (2-AG and AEA) contributions to DMS-mediated behavioral flexibility and synaptic transmission." - The organization and presentation of results are confusing, with key findings difficult to follow. Many results are relegated to supplementary figures, making it challenging to assess methods and conclusions. Important variables like sex differences and tracking behaviors are collapsed, obscuring the effects. The paper should consistently emphasize these variables in analyses and figures.

We moved the majority of figures to the main text and changed the order of graphs so that there is a systematic analysis of behavior for tracking and sex differences. We present behavioral data in a more systematic way, first collapsed across all variables, then split by tracking, then by sex. We have emphasized key findings throughout the manuscript to aid in assessment of results and conclusions. In Figure 4, we updated the analyses to include Tracking and Sex for post-devaluation consumption data (Fig. 4A-B) and non-sated, non-reinforced PLA tests (Fig. 4C-D). Because a smaller subset of rats were run in the within-subject rimonabant dose response (Fig. 4E-F) on reinforced PLA test (i.e. insufficient power for tracking), we only present lever data split by sex (reporting n's), which was the only significant effect observed in that analysis.

- The exclusive focus on inhibitory currents is surprising given CB1R's role in glutamatergic transmission. The lack of excitatory current data limits interpretation.

We clarified our rationale for focusing on inhibitory currents. This is due to our behavioral finding that DMS CB1R signaling (inhibition of synaptic transmission) promotes Pavlovian devaluation sensitivity, running counter to an established mechanism of CB1R-mediated inhibition of glutamatergic transmission that prevents instrumental devaluation sensitivity. (See response to major concern 1). We provide context for our focus on inhibitory synaptic transmission based on the canonical model of DMS function to promote behavioral flexibility.

- The synaptic physiology findings are not well linked to the observed behavioral outcomes.

We have added text linking these findings to the discussion.

Discussion (line 546): "Our in vivo behavioral pharmacology experiment revealed sex differences in devaluation sensitivity, in which male rats were flexible while female rats are not. Consistent with our DMS disinhibition hypothesis, we found that male rats showed reduced DMS inhibitory synaptic transmission in our ex vivo slice electrophysiology experiments. This supports the idea that under vehicle conditions, intact CB1R signaling reduces inhibitory synaptic transmission, potentially promoting DMS activation and flexible responding in male rats. Additionally, we find that male rats require DMS CB1R signaling to express flexible responding. In DMS slices, we saw evidence for CB1R-mediated inhibition of GABAergic synaptic transmission in male rats. While not directly tested, this suggests a viable mechanism by which rimonabant infusions block DMS disinhibition and impair "goal-directed" Pavlovian devaluation sensitivity in male rats. There are many possibilities that remain untested regarding sex-differences in DMS CB1R regulation of behavioral flexibility: (1) males may express more DMS CB1Rs (2) males may have enhanced DMS CB1R function or (3) outcome devaluation procedures may result in greater eCB release in males than females. Our slice electrophysiology studies suggest that DMS CB1R expression does not differ between males and females as both sexes are sensitive to CB1R activation, though a full dose response for CB1R agonist would be needed to rule out this possibility. Downstream of the CB1R in other brain regions, CB1R function and intracellular signaling differs between males and females (Tabatadze et al., 2015), and this may be the case in DMS. Upstream of CB1R signaling, males and females may differ in DMS eCB release dynamics, though this has yet to be investigated." - The lack of consistency in food types used for devaluation (pellets vs. chow) complicates interpretation of consumption data.

It is true that the two outcomes (pellets and chow) are not as well matched as they are in some studies (i.e. 2 types of 45 mg pellets). However, we are using identical satiety procedures as in prior devaluation studies (four studies referenced in lines 181-182). For any given choice test, outcomes are counterbalanced. The consumption data are presented accordingly, as sated outcome (pellets or chow) vs. non-sated outcome (chow or pellets). Thus, the outcomes are counterbalanced within each devaluation and choice test leading to limited interpretational pitfalls.

- Differences in food deprivation protocols between behavioral and electrophysiology experiments could affect basal cannabinoid activity and outcomes.

We included consideration of this caveat in the discussion section.

Discussion text (line 568): "Additionally, food restriction levels interact with schedules of reinforcement to control task engagement in outcome devaluation and may also influence DMS engagement and associated neurophysiology (Chevée et al., 2023). Recent studies show that even short-term food restriction alters DS physiology (Campanelli et al., 2021). While no studies to date correlate the length of food restriction with changes to DMS physiology specifically, it is possible that the limited food restriction procedure in our slice electrophysiology experiments limited detection of sex differences or obscured other DMS physiology changes that we may have seen with longer restriction time periods used in behavioral studies." - The use of different doses of rimonabant is not clearly presented, raising questions about dose effects.

We have added text to clarify our use of rimonabant doses within the context of the literature.

Methods text (line 171): "Rimonabant shows a high affinity for CB1Rs and exerts measurable effects on behavior at doses including 1µg/µL and lower, as shown in prior work (Oleson et al., 2012; Wenzel and Cheer, 2018)." Results text (line 398): "Rimonabant shows a high affinity for CB1Rs and exerts measurable receptor effects at lower doses including 1µg/µL, as shown in prior work (Wenzel and Cheer, 2018). We used the low dose (1µg/µL) of rimonabant for outcome devaluation tests and observed behavioral effects at this dose that did not impact behavior in either reinforced or non-reinforced PLA sessions or consumption during pre- or post-tests." - Small group sizes may limit the power to detect significant effects, requiring cautious interpretation of null results.

It is not clear which null results are considered underpowered by reviewers, so we address a few possibilities below and in the revised text. Perhaps the lack of devaluation sensitivity in female rats is of concern, particularly looking at females within tracking groups. We have revised our results reporting for Fig. 3 and Supplemental Figs. 3-1 and 3-2, noting analyses may be underpowered and adding effect sizes for each statistic reported. An example of this is found below that is representative of many similar revisions in main text:

Results (line 338): "While potentially underpowered within tracking/sex groups, post-hoc analyses confirmed that under vehicle conditions male ST rats were sensitive to devaluation (t(7)=3.910, p=0.006, Cohen's D = 1.38), while intra-DMS rimonabant injections impaired devaluation sensitivity with similar levels of Pavlovian approach for valued and devalued conditions (t(5)= -0.556, p=0.602, Cohen's D =-0.26)." Results (line 350): "Notably, null effects should be interpreted with caution due to low sample sizes within tracking/sex groups." Regarding female devaluation performance, our findings are consistent with several studies, yet it is possible females would be sensitive in other measures not captured in our task. We have added text to this effect in the discussion.

Discussion (line 468): "However, null devaluation effects for females should be interpreted with caution, particularly within specific tracking groups as we were less powered to detect effects in those analyses. It is also possible there are other behaviors (i.e. conditioned orienting, post-CS responding, etc.) that are not measured here for which females may express behavioral flexibility (Sood and Richard, 2023)." Notably, for null effects on sIPSC amplitude, for the same cells we detected effects on sIPSC frequency, suggesting these analyses are powered to detect differences, and point to presynaptic vs. post-synaptic mechanisms of action.

Minor Concerns - Awkward phrasing and tense changes throughout the manuscript decrease readability.

We updated the language used throughout the manuscript for consistency. Notably, as is commonly practiced in scientific manuscripts, we use present tense in the Introduction and Discussion sections, particularly as it relates to discussion of prior studies and interpretations/conclusions from the current study. Across all sections, we use the past tense in the reporting of the current studies methods, analyses and results.

- The introduction suggests that the amount of conditioned responding is related to sensitivity to outcome devaluation (paraphrasing for space but the sentence - "Female rats show more sign-tracking, so they may be less sensitive"). Is this true or has this been demonstrated in this study or others? We have updated the text to clarify within the context of prior studies.

Intro text (line 97): Female rats show more lever-directed approach during PLA and are more likely to be characterized as ST rats compared to males (Hammerslag and Gulley, 2014; Keefer et al., 2022; King et al., 2020; Kochli et al., 2020; Madayag et al., 2017; Pitchers et al., 2015), suggesting they may be less sensitive to outcome devaluation even after extended training. Indeed, in some studies examining sex differences, females are less sensitive to instrumental and Pavlovian devaluation (Bien and Smith, 2023; Quinn et al., 2007; Schoenberg et al., 2019; Sood and Richard, 2023).

- Abbreviations are used inconsistently, reducing accessibility for readers.

We have fixed abbreviations for consistency.

- Clarification is needed for the use of Bonferroni correction in statistical tests.

We included rationale for using Bonferroni correction in the Methods section.

Methods (line 254): "We used Bonferroni correction when performing multiple statistical tests simultaneously on a subset of appropriate pairwise comparisons (i.e. comparing responding in Valued vs. Devalued within each group, but not comparing Devalued between groups).

- The description of lever delivery should use "variable time" instead of "variable interval" to accurately describe the procedure.

We have updated this wording in the Methods section.

- Sex differences in electrophysiology data should be consistently reported, even if no differences are observed with the drug.

We included the statistical analysis of pre- and post-WIN application for both males and females in the results.

- The manuscript should include simple effects (devaluation effects for each group) to support conclusions drawn.

For all meaningful interactions we now include simple effects, including devaluation effects for each group presented. As indicated above, we indicate when we are underpowered and caution interpretation.

- The authors should rethink what is included as extended vs. primary data.

We have updated figure order and included most figures in the main text.
